# Immune profile of patients with peritoneal carcinomatosis selected for CRS-HIPEC therapy

**DOI:** 10.1007/s00262-023-03515-2

**Published:** 2023-08-14

**Authors:** Julia Kleber, Jordi Yang Zhou, Florian Weber, Florian Bitterer, Patricia Hauer, Paul Kupke, Katharina Kronenberg, Edward K. Geissler, Hans J. Schlitt, Matthias Hornung, James A. Hutchinson, Jens M. Werner

**Affiliations:** 1 Department of Surgery, University Hospital Regensburg, Franz-Josef-Strauss-Allee 11, 93053 Regensburg, Germany; 2 Leibniz Institute for Immunotherapy, Regensburg, Germany; 3 Institute for Pathology, University of Regensburg, Regensburg, Germany

**Keywords:** Peritoneal carcinomatosis, Colorectal cancer, CRS/HIPEC, Immunotherapy, Memory *T* cells

## Abstract

Cytoreductive surgery (CRS) combined with hyperthermic intraperitoneal chemotherapy (HIPEC) is a treatment option for peritoneal carcinomatosis (PC) from colorectal cancer (CRC), which is otherwise a terminal stage of disease. Nevertheless, survival outcomes are only marginally superior to other treatments. This fact highlights the need for better strategies to control intra-abdominal disease recurrence after CRS-HIPEC, including the complementary use of immunotherapies. The aim of this study was therefore to investigate the immune phenotype of *T* cells in patients with PC. Fifty three patients with CRC (34 patients with PC and 19 patients without PC) were enrolled in a prospective study (clinicaltrials.gov: NCT04108936). Peripheral blood and omental fat were collected to isolate peripheral blood mononuclear cells (PBMCs) and adipose tissue mononuclear cells (ATMCs). These cells were analysed by flow cytometry using a panel focused upon *T* cell memory differentiation and exhaustion markers. We found a more naïve profile for CD8^+^ *T* cells in peripheral blood and intra-abdominal fat of PC patients compared to comparator group (CG) patients. Furthermore, there was an over-representation of CD4^+^ *T* cells expressing inhibitory receptors in adipose tissue of PC patients, but not in blood. Our description of intraperitoneal *T* cell subsets gives us a better understanding of how peritoneal carcinomatosis shapes local immune responses.

**Supplementary Information:**

The online version contains supplementary material available at 10.1007/s00262-023-03515-2.

## Introduction

Peritoneal carcinomatosis (PC) carries a poor prognosis and was considered as a terminal stage of disease until the introduction of cytoreductive surgery followed by hyperthermic intraperitoneal chemotherapy. Cytoreductive surgery (CRS) is a radical procedure that aims to completely resect all intra-abdominal tumour tissue. Hyperthermic intraperitoneal chemotherapy (HIPEC) involves local delivery of high-dose chemotherapeutics in warmed solutions with the intention of optimizing tumouricidal activity and minimizing systemic toxicity. Although CRS-HIPEC is regarded a promising treatment for patients with PC secondary to gastrointestinal [1] or ovarian cancer [2] without extra-abdominal disease, there remain unanswered questions about eligibility criteria and effectiveness of CRS-HIPEC beyond highly selected patient groups [3].

Patient selection for CRS-HIPEC is important for achieving complete cytoreduction, hence a useful therapeutic effect. Apart from the absence of widespread tumour dissemination or tumour complicated with obstruction or perforation, current guidelines emphasize patients’ fitness for therapy as a key eligibility criterion. Only patients with an ECOG (Eastern Cooperative Oncology Group) performance status of ≤ 2 points should be considered to receive a CRS-HIPEC procedure, which generally selects for younger patients [4].

Even if acceptable rates of mortality and morbidity are achievable through narrow patient selection, survival outcomes with CRS-HIPEC may only be marginally superior to other surgical approaches such as CRS alone [5]. The need for better strategies to control intra-abdominal disease recurrence after CRS-HIPEC has sparked interest in possible combination therapies, such as anti-PD-1 or other immune checkpoint inhibitors (ICIs) [6, 7]. A better understanding of *T* cell immunity in patients with widespread intraperitoneal metastases could lead to a more rational approach to immunotherapy for PC. Hence, in this study, we investigated the immune phenotype of *T* cells isolated from peripheral blood and omental fat of patients with PC secondary to colorectal carcinoma and compared these to patients with advanced colorectal carcinoma but without PC.

## Materials and methods

### Study design

A prospective, single-centre observational study was conducted with approval of the Ethics Committee of the University of Regensburg (15-101-0357). The study was registered with the Deutsche Krebsgesellschaft (StudyBoxNumber ST-U091) and ClinicalTrials.gov (NCT04108936). Inclusion criteria were patients with colorectal carcinoma (CRC) with or without peritoneal carcinomatosis (synchronous or metachronous) and age > 18 years. Between 2016 and 2021, 53 patients consented to participate. Thirty-four patients had PC from CRC, 15 synchronous, and 19 metachronous (Table 1). Nineteen patients had CRC with loco-regional disease but without PC or distant metastasis as a comparator group (CG). On the day of surgery, peripheral blood was collected preoperatively and samples of intra-abdominal fat from the omentum majus were harvested during standard-of-care surgical procedures. Tumours in the peritoneal cavity often metastasize first to the omentum, then throughout the abdomen before appearing in ascites. In the omentum, there are aggregates of leucocytes, known as milky spots or fat-associated lymphoid clusters (FALCs), which are embedded between adipocytes just beneath peritoneal mesothelial cells. Milky spots filter the peritoneal fluid, making them ideal locations to generate immune responses to any sort of antigens or pathogens in the peritoneal cavity [8].

**Table 1:** Summary of patient characteristics

	Case		Control		*p*-value
	*n*	%	*n*	%	
Sex					
Male	13	38.2%	16	84.2%	0.0016^1^
Female	21	61.8%	3	15.8%	
Age					
Mean (SD)	56 (± 12)		69 (± 10)		0.0002^2^
Median (IQR)	56 (± 16)		68 (± 9)		
Range	28–82		52–88		
Staging					
Tis	1	3.0%	0	0.0%	
Tx	1	3.0%	0	0.0%	
T0	0	0.0%	1	5.3%	
T1/2	0	0.0%	6	31.6%	
T3/4	25	73.5%	10	52.6%	
unknown	7	20.5%	2	10.5%	
N0	7	20.6%	12	63.2%	
N1	8	23.5%	3	15.8%	
N2	7	20.6%	1	5.2%	
unknown	12	35.3%	3	15.8%	
*Grading*					
G1	4	11.8%	6	31.6%	
G2	8	23.5%	10	52.6%	
G3	10	29.4%	0	0.0%	
unknown	12	35.3%	3	15.8%	
*Resection status (case: CCR; control: R)*					
0	14	41.2%	19	100%	
1	14	41.2%	0	0.0%	
2	6	17.6%	0	0.0%	
*Peritoneal cancer index*					
< 20	30	88.2%	not applicable		
> 20	4	11.8%			
*Preoperative chemotherapy*					
Yes	11	32.4%	2	10.5%	0.1024^1^
No	23	67.6%	17	89.5%	
*Microsatellite status*					
Stable	24	70.6%	14	73.7%	0.7292^1^
Instable	6	17.6%	5	26.3%	
Unknown	4	11.8%	0	0.0%	
*Timepoint of peritoneal carcinomatosis*					
Synchronous	15	44.1%	not applicable		
Metachronous	19	55.9%			

*SD* Standard deviation, *IQR* Interquartile range

Staging depending on size and invasive growth. Grading depending on cellular differentiation. Resection status after surgical intervention: *CCR* Completeness of cytoreduction (0 = no visible residual disease; 1 = residual disease less than 2.5 mm; 2 = residual disease more than 2.5 mm). *R* Resection (0 = no residual tumour; 1 = microscopic residual tumour; 2 = macroscopic residual tumour). Peritoneal Cancer Index (PCI) depending on topographic dissemination. https://doi.org/10.1007/978-1-4613-1247-5_23

^1^Fisher’s exact test

^2^2-tailed *t*-test

### Sample processing

Peripheral blood mononuclear cells (PBMCs) were isolated by Ficoll density gradient centrifugation. Adipose tissue mononuclear cells (ATMCs) were isolated from intra-abdominal fat samples, which were first dissected then dissociated into a single cell suspension using a GentleMACS device (Miltenyi). Samples were digested in 2 ml RPMI medium (Gibco) supplemented with 20 U/ml CLSPA (Worthington) for every 4 g fat, over 1 h at 37 °C. The resulting cell suspension was washed in RPMI, and the fat layer that formed after centrifugation was decanted before the cell pellet was resuspended and filtered through a 40-µm nylon mesh. The cell suspension was then separated by Ficoll density gradient centrifugation. Isolated PBMCs and ATMCs were stored in cryopreservation medium at 10^6^ cells/100 µl [70% FCS (Gibco), 20% RPMI, 10% DMSO] in liquid nitrogen.

### Flow cytometry

Cells were thawed and 200 µl of cell suspension per well was transferred into 96-well plates. The cells were washed once with 200 µl Cell Staining Buffer (CSB from Biolegend) at 1400 rpm for 5 min. Cells were resuspended in 15 µl CSB supplemented with 15% FCR Block (Miltenyi) and incubated for 10 min at 4 °C. Fifty microliter of antibody mastermix for extracellular staining was then added to each well (Fig. S1). Samples were incubated for 30 min at 4 °C before being washed twice with 200 µl CSB. Finally, cells were filtered through a nylon mesh then were fixed in 200 µl of PBS supplemented with 0.5% IOTest3 (Beckman Coulter). As previously described, data were collected with a CytoFlex LX instrument (Beckman Coulter) and were subsequently analysed in Kaluza and CytoBank (Beckman Coulter) [9]. FlowSOM analysis was performed by applying an automated approach of clustering and visualization algorithms based on self-organizing maps [10].

### Statistics

Mann–Whitney tests with Benjamini–Hochberg adjustment for multiple testing with a false-discovery rate (FDR) of 5% were used for significance testing of cell subset frequencies in PC and CG patients (IBM SPSS Statistics v28). Discriminatory features were evaluated by calculating the area-under-the-curve (AUC) of receiver operating characteristic (ROC) curves. Fisher’s exact test or a 2-tailed *t*-test was used for all tests of significance. Pearson *R*^2^ was used as a measure of bivariate linear correlation. All plots were generated using GraphPad Prism (GraphPad Prism v9.4.0).

## Results

### Immunophenotypic differences in PBMCs associated with PC patients

**Fig. 1:**
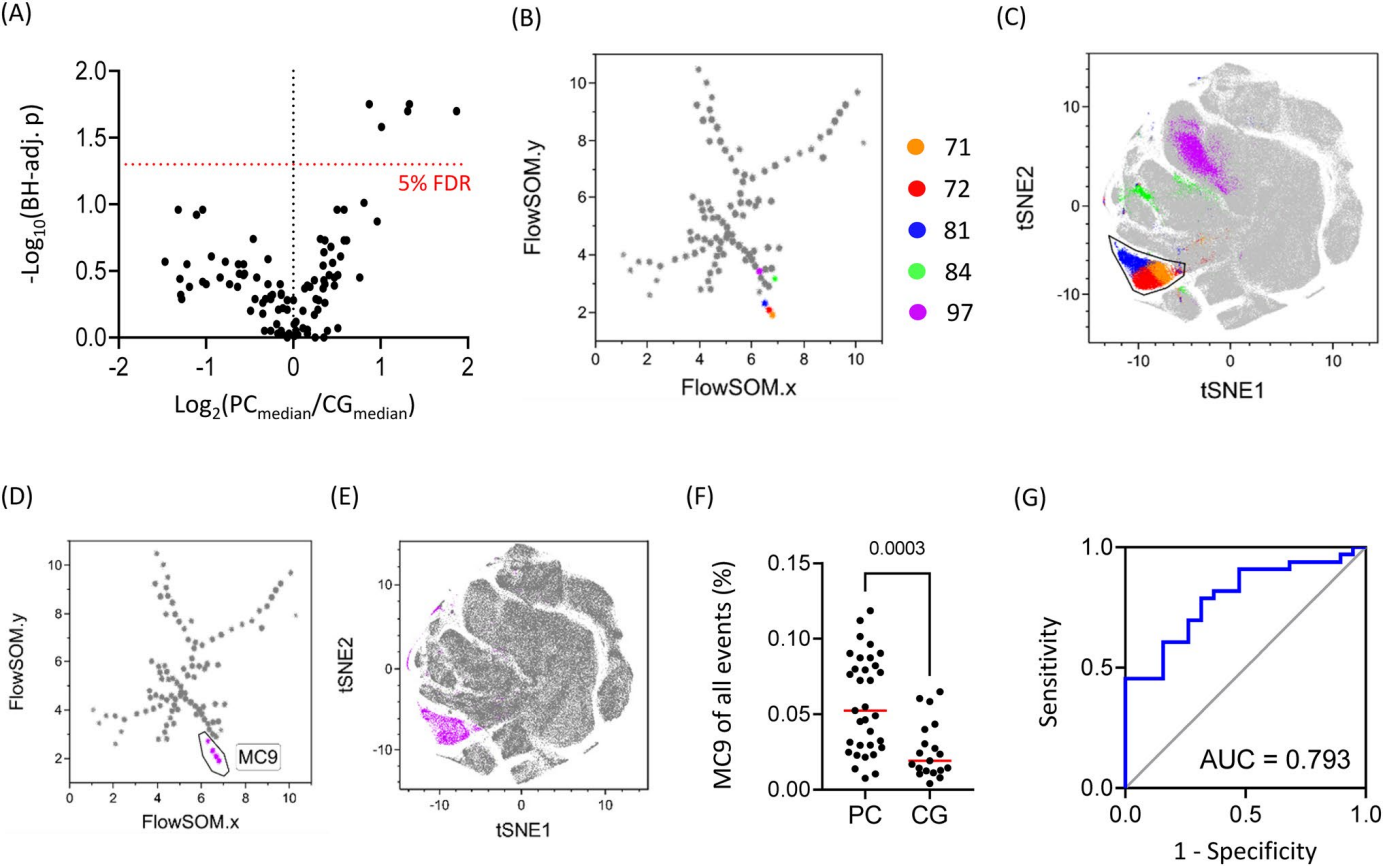
Comparative immune profiling of peripheral blood *T* cells. Flow cytometry analysis of PBMCs was performed using samples from *n* = 34 patients (PC) and *n* = 19 patients (CG). **A** *T* cell subpopulations were defined using FlowSOM, an unsupervised clustering algorithm. Differentially represented cell clusters in PC and CG patients were discovered using Mann–Whitney tests with Benjamini–Hochberg correction (FDR = 5%). **B** FlowSOM tree showing 5 differentially represented cell clusters. **C** Projection of 5 differentially represented cell clusters to a tSNE plot, demonstrating the close phenotypic similarity of clusters 71, 72, 81, 84, and 97. **D** FlowSOM tree showing that 3 significantly differentially represented clusters 71, 72, and 81, as well as cluster 61 contributed to metacluster 9 (MC9). **E** Projection of MC9 cells to a tSNE plot. **F** MC9 cell frequencies in PC and CG patients (MW test; *p* = 0.0003). **G** ROC curve demonstrating the discriminatory value of MC9 as a marker of PC patients

We first investigated whether patients with peritoneal carcinomatosis (PC) secondary to colorectal carcinoma could be distinguished from comparator group (CG) patients, who had colorectal carcinoma but not PC, by immunophenotyping of peripheral blood *T* cells. PBMC samples from PC and CG patients were analysed by flow cytometry using an antibody panel focused on *T* cell memory differentiation and exhaustion markers (Fig. S1). Data were analysed by first performing an unsupervised clustering of cells using FlowSOM, then comparing the frequencies of cells in clusters between PC and CG patients (Fig. 1A). Five closely related, differentially represented cell clusters were identified (Fig. 1B). Three of five clusters showed particular phenotypic similarity (Fig. 1C) and primarily accounted for metacluster 9 (Fig. 1D, E). Metacluster 9 (MC9) was represented in PC and CG patients (Fig. 1F), reaching statistic differences and was a good discriminatory marker of these subgroups (Fig. 1G). Notably, MC9 cell frequencies were not associated with patient sex (Fig. S2A). However, patients who received preoperative chemotherapy exhibited lower frequencies of cells in MC9 (Fig. S2B) and MC9 cell frequencies inversely correlated with patient age (Fig. S2C, D).

**Fig. 2:**
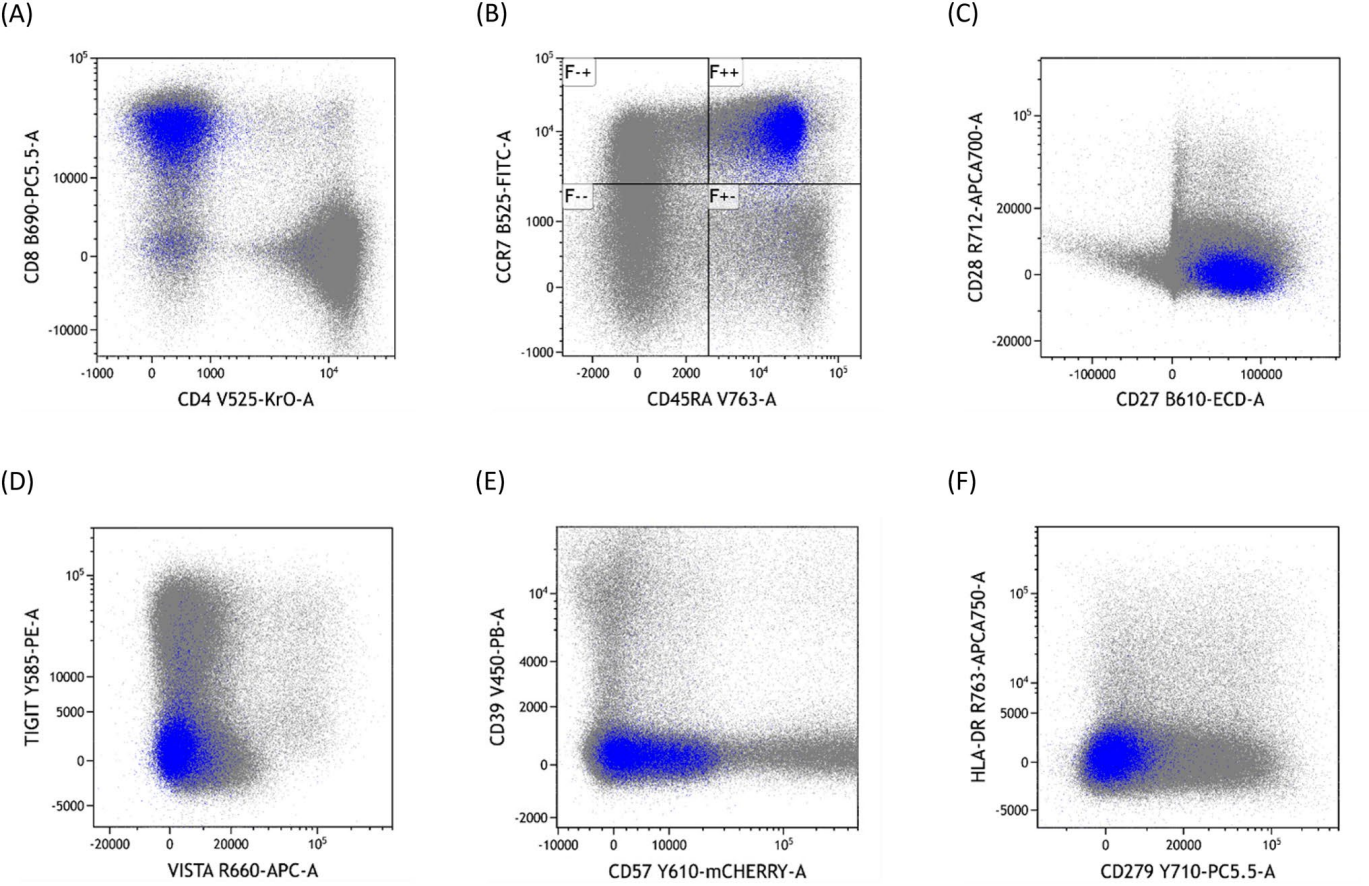
Phenotypic definition of peripheral blood *T* cells contributing to MC9. Combined dataset from n = 39 PC patients comparing MC9 cells (blue) and all other events (grey). **A** Plot of CD4 versus CD8 expression. **B** Plot of CD45RA versus CCR7 expression. **C** Plot of CD27 versus CD28. **D** Plot of VISTA versus TIGIT. **E** Plot of CD57 versus CD39. **F** Plot of CD279 versus HLA-DR

To identify the cell types circumscribed by MC9, we mapped MC9 onto manually gated flow cytometry data from PBMCs. MC9 cells were predominantly unactivated CD8^+^ CD45RA^+^ CCR7^+^ naïve *T* cells that broadly lacked expression of CD28, TIGIT, VISTA, CD39, CD57, HLA-DR, and PD-1 (CD279) (Fig. 2A-F). Hence, PC patients exhibited higher circulating frequencies of naïve CD8^+^ *T* cells.

### Immunophenotypic differences in ATMCs associated with PC patients

**Fig. 3:**
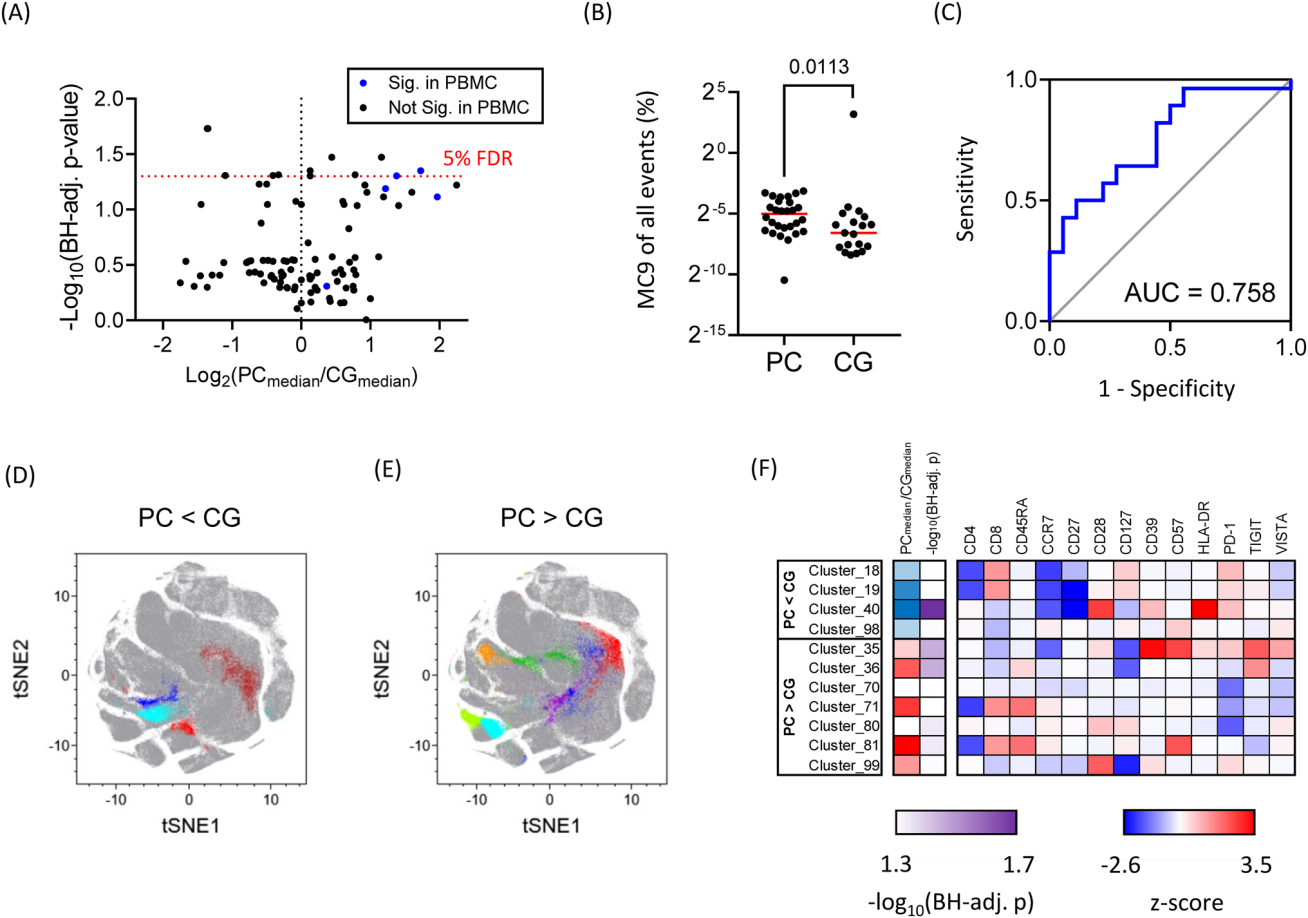
Comparative immune profiling of omental fat *T* cells. Flow cytometry analysis of ATMCs was performed using aggregated samples from *n* = 28 patients (PC) and *n* = 18 patients (CG). **A** Differentially represented cell clusters in PC and CG patients were discovered using Mann–Whitney tests with Benjamini–Hochberg correction (FDR = 5%). **B** MC9 cell frequencies in PC and CG patients (MW test; p = 0.0113). **C** ROC curve demonstrating the discriminatory value of MC9 as a marker of PC patients. **D** Projection of under-represented clusters 18, 19, 40, and 98 to a tSNE Plot (PC versus CG). **E** Projection of over-represented clusters 35, 36, 70, 71, 80, 81, and 99 to a tSNE Plot (PC versus CG). **F** Presentation of statistical significance after Benjamini–Hochberg correction and phenotypic analysis of over- and under-represented single clusters (PC versus CG). Red-coloured boxes correspond with increased marker expression; blue with decreased

We next asked whether the cell subset distribution associated with PC in PBMC samples was reflected in adipose tissue mononuclear cell (ATMCs) samples. Flow cytometry data were captured from ATMC samples with the same methods used for PBMC analysis. The same FlowSOM clustering from PBMC samples was applied to ATMCs in order to identify differentially represented cell subsets. Notably, clusters 71 and 81 that contribute to MC9 were over-represented in ATMC samples (Fig. 3A). MC9 was also significantly over-represented in PC versus CG samples (Fig. 3B) and was a good discriminatory marker of patient subgroups (Fig. 3C). Phenotypic analysis of MC9 cells from ATMCs confirmed they were naïve CD8^+^ *T* cells (Fig. S3). Thus, over-representation of MC9 in adipose tissue seems to be a consistent immunological feature of PC patients.

Besides clusters 71 and 81, we observed a relative over-representation of clusters 35, 36, 70, 80, and 99 in PC patients; in addition, clusters 18, 19, 40, and 98 were under-represented (Fig. 3D, E). Of special interest, clusters 35 and 36 represented activated CD4^+^ memory *T* cells (*T*_mem_) with elevated expression of inhibitory receptors, including PD-1, TIGIT, and VISTA (Fig. 3F & S4). Over-representation of these *T*_mem_ in ATMCs from PC versus CG patients, and only in fat, not in blood samples hints at a possible tumour-driven immune response leading to *T* cell exhaustion, anergy, or regulation [11].

## Discussion

Here, we compared immune phenotypes between patients with PC from CRC and patients with loco-regional CRC without distant metastasis. We made two interesting immunological observations about these two groups of patients: First, PC patients had a higher frequency of naïve CD8^+^ *T* cells compared to CG patients both in peripheral blood and omental adipose tissue. And second, PC patients had a CD4^+^ *T*_mem_ subset of adipose tissue-derived *T* cells that expressed elevated levels of PD-1, TIGIT, and VISTA.

We observed a significant over-representation of naïve CD8^+^ *T* cells in peripheral blood and omental fat in PC patients compared to non-PC patients. Notably, PC patients selected for CRS-HIPEC were generally younger than CG patients. Age-related decline of naïve CD8^+^ *T* cell frequency is a well-described phenomenon in healthy and diseased people [12]. Therefore, selection of PC patients according to fitness for surgery, which biases towards younger patients, is a likely explanation for this finding. Nevertheless, a more naïve *T* cell profile may be favourable in terms of response to immunotherapy [13].

A third of our PC patients received preoperative chemotherapy and all of them were metachronous PC. These patients were treated according to guidelines for management of colorectal cancer so that they received folinic acid, 5-fluorouracil, oxaliplatin or irinotecan (FOLFOX/ FOLFOXIRI). Some patients additionally received bevacizumab, a therapeutic antibody against vascular endothelial growth factor, to inhibit neovascularization. We cannot exclude that prior chemotherapy, rather than tumour-related effects, might account for some of the immunological differences observed in our study [14] since temporal changes have been reported in the peripheral immune cell composition and cytokine production in response to chemoradiation therapy in rectal cancer patients. Especially, the proportion of CD4^+^ *T* cells among total lymphocytes was relatively higher than that of CD8^+^ *T* cells during chemoradiation therapy. However, after treatment termination, the proportion of CD8^+^ *T* cells increased and was similar to the proportion of CD4^+^ *T* cells [15].

We speculate that the over-representation of CD4^+^ *T*_mem_ cells expressing inhibitory receptors from adipose tissue of PC patients reflects tumour-driven *T* cell exhaustion [16]. In particular, we discovered CD4^+^ *T*_mem_ over-expressing PD-1, TIGIT, and VISTA. Therefore, these receptors might represent valuable therapeutic targets in PC patients. Defining an approach to address these targets is challenging. In particular, systemic drug administration has the disadvantage of having limited access to the abdominal compartment and the possibility of producing systemic toxicity [17]. Therefore, the direct administration of immunotherapies into the peritoneal cavity represents an interesting strategy.

So far, catumaxomab had been the only in Europe approved monoclonal antibody used for intraperitoneal application and treatment of malignant ascites. Catumaxomab is a trivalent antibody that crosslinks CD3^+^ T cells with epithelial cell adhesion molecule (EpCAM) expressing tumours in the presence of FcR-bearing myeloid antigen presenting cells, which primes cellular and humoral responses against tumour antigens [18]. Beneficial effects of catumaxomab cotreatment were reported in several studies in patients with a range of tumour entities [19]. Of interest, Ströhlein et al*.* [20] showed an acceptable safety profile for intraperitoneal use of catumaxomab in patients with PC secondary to colon, gastric, and pancreatic cancer . Recent investigations in murine models of PC provide encouraging preclinical results for intraperitoneal immunotherapy [21]. However, catumaxomab was voluntarily withdrawn due to commercial reasons. Another immunotherapy approach is the treatment with Chimeric Antigen Receptor (CAR)-T cells. CAR-T cell therapy was first used in haematological malignancies and obtained promising results. This led to the development of CAR-T cells for targeting solid tumours. However, their use in solid tumour and their efficacy have not at all achieved the expected results [22]. Second-generation CAR-T cells targeting CEA to treat peritoneal carcinomatosis have been used in murine model, demonstrating that local peritoneal infusion of CAR-T cells was superior to systemic administration [23]. Furthermore, using a PC mouse model of MC38 colon cancer, it has been shown that intraperitoneal immunotherapy with oncolytic vaccinia virus is able to restore peritoneal anticancer immunity and potentiate immune checkpoint blockade to suppress PC and malignant ascites [24].

In conclusion, our study revealed a more naïve profile for CD8^+^ *T* cells in peripheral blood and omental fat of PC patients. More importantly, we discovered an over-representation of CD4^+^ memory *T* cells expressing inhibitory receptors in omental fat of PC patients, but not in their blood or adipose tissue of non-PC patients, which suggests local anti-tumour immunity might be compromised. The favourable systemic immune profile of PC patients leads us to the proposition that intraperitoneal application of therapeutic antibodies against PD-1, TIGIT, or VISTA could enhance their local efficacy whilst minimizing systemic toxicity.

### Supplementary Information

Below is the link to the electronic supplementary material.

Supplementary file1 (PDF 1782 KB)

## Data Availability

The datasets generated during and/or analysed during the current study are available from the corresponding author on reasonable request.
