# Supplementary material for: Immune profile of patients with peritoneal carcinomatosis selected for CRS-HIPEC therapy
Source: Cancer Immunol Immunother. 2023 Aug 14;72(11):3867–73. doi: 10.1007/s00262-023-03515-2 (PMC10576707; doi:10.1007/s00262-023-03515-2)
Supplement: Supplementary file 1 — Supplementary file1 (PDF 1782 KB) [file 262_2023_3515_MOESM1_ESM.pdf]

	BUV395	BUV661	KB520	ECD	PerCP	PE	AF594	PC5.5	PC7	AF647	AF700	AF750	BV421	BV510	BV785	Viability
**Antigen**	**CD45**	**CD3**	**CCR7**	**CD27**	**CD8**	**TIGIT**	**CD57**	**CD279**	**CD127**	**VISTA**	**CD28**	**HLA-DR**	**CD39**	**CD4**	**CD45RA**	Live/Dead
Clone	HI30	UCHT1	G043H7	1A4CD27	SK1	A15153G	HNK1	PD1.3	A019D5	MIH65.rMAb	CD28.2	Immu-357	A1	RPA-T4	HI100	
Isotype	mIgG1	mIgG1	mIgG2a	mIgG1	mIgG1	mIgG2a	mIgM	mIgG2b	mIgG1	mIgG1	mIgG1	mIgG1	mIgG1	mIgG1	mIgG2b	
Fluorochrome	BUV395	BUV661	KB520	ECD	PerCP	PE	AF594	PC5.5	PC7	AF647	AA700	AA750	BV421	BV510	BV785	ViaKr-808
Vol. (µl)	2.5	2,0	2.5	4,0	1.5	5,0	0.6	4.5	5,0	5,0	2.0	2.0	5	2.0	2.5	1,0
Supplier	BD	BD	BioLegend	BC	BioLegend	BioLegend	BioLegend	BC	BioLegend	BD	BC	BC	BioLegend	BioLegend	BioLegend	BC
Cat.#	563792	612964	353260	B26603	344708	372714	359626	B36123	351320	749646	B12696	B42021	328214	300546	304140	C36628
Status	RUO	RUO	RUO	ASR	RUO	RUO	RUO	ASR	RUO	RUO	ASR	ASR	RUO	RUO	RUO	RUO

Figure S1 Extracellular 16-colour antibody panel for flow cytometry analysis.

T cell memory differentiation and exhaustion markers.

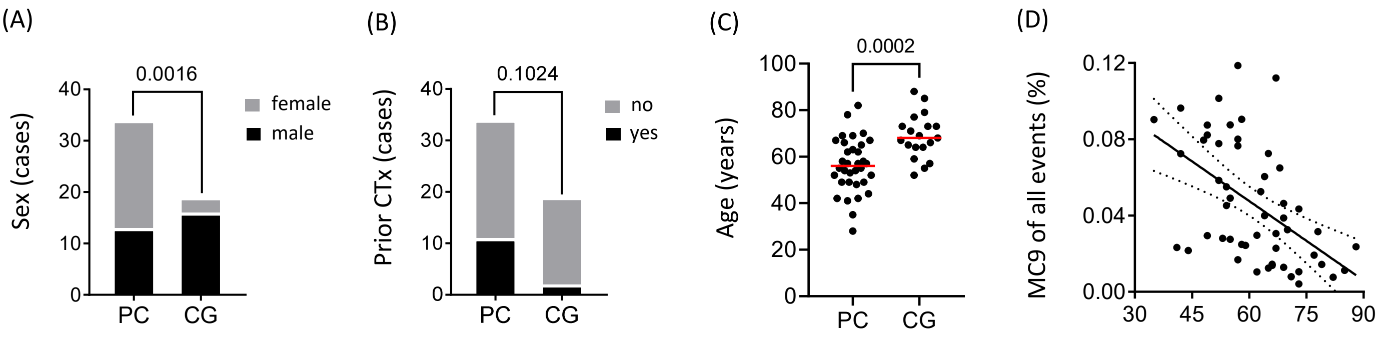

Figure S2 Analysis for potential co-variables

(A) Equal representation of males and females in the PC and CG patient groups (Fisher's exact test). **(B)** Predominance of patients who received prior adjuvant chemotherapy (Prior CTx) in PC versus CG patients (Fisher's exact test). **(C)** Patient ages in PC and CG groups (MW test). **(D)** MC9 cell frequencies correlated with patient age (Pearson $R^2 = 0.265$, $p < 0.0001$).

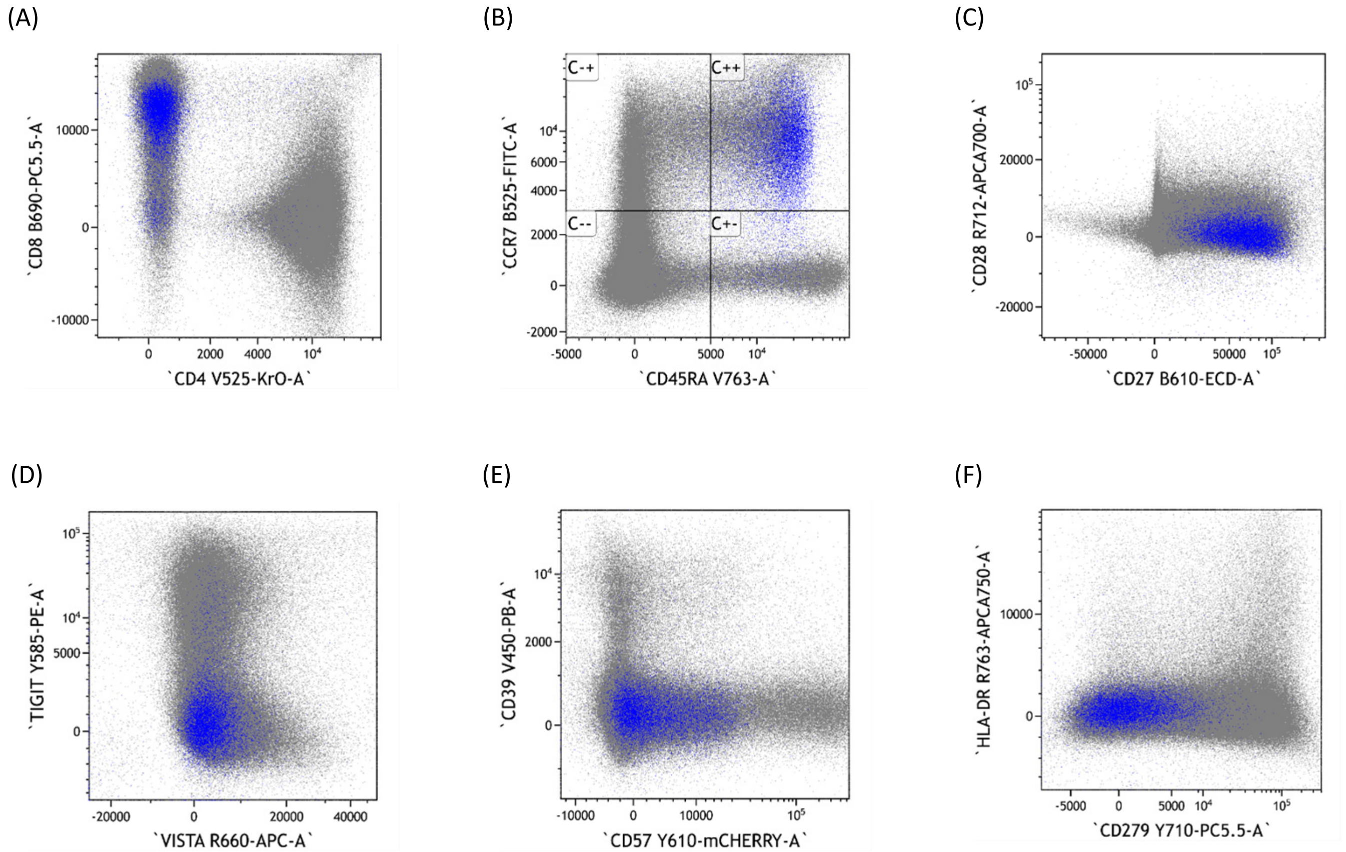

Figure S3 Phenotypic definition of omental fat T cells contributing to MC9. Combined dataset from n=34 PC patients comparing MC9 cells (blue) and all other events (grey). **(A)** Plot of CD4 versus CD8 expression. **(B)** Plot of CD45RA versus CCR7 expression. **(C)** Plot of CD27 versus CD28. **(D)** Plot of VISTA versus TIGIT. **(E)** Plot of CD57 versus CD39. **(F)** Plot of CD279 versus HLA-DR.

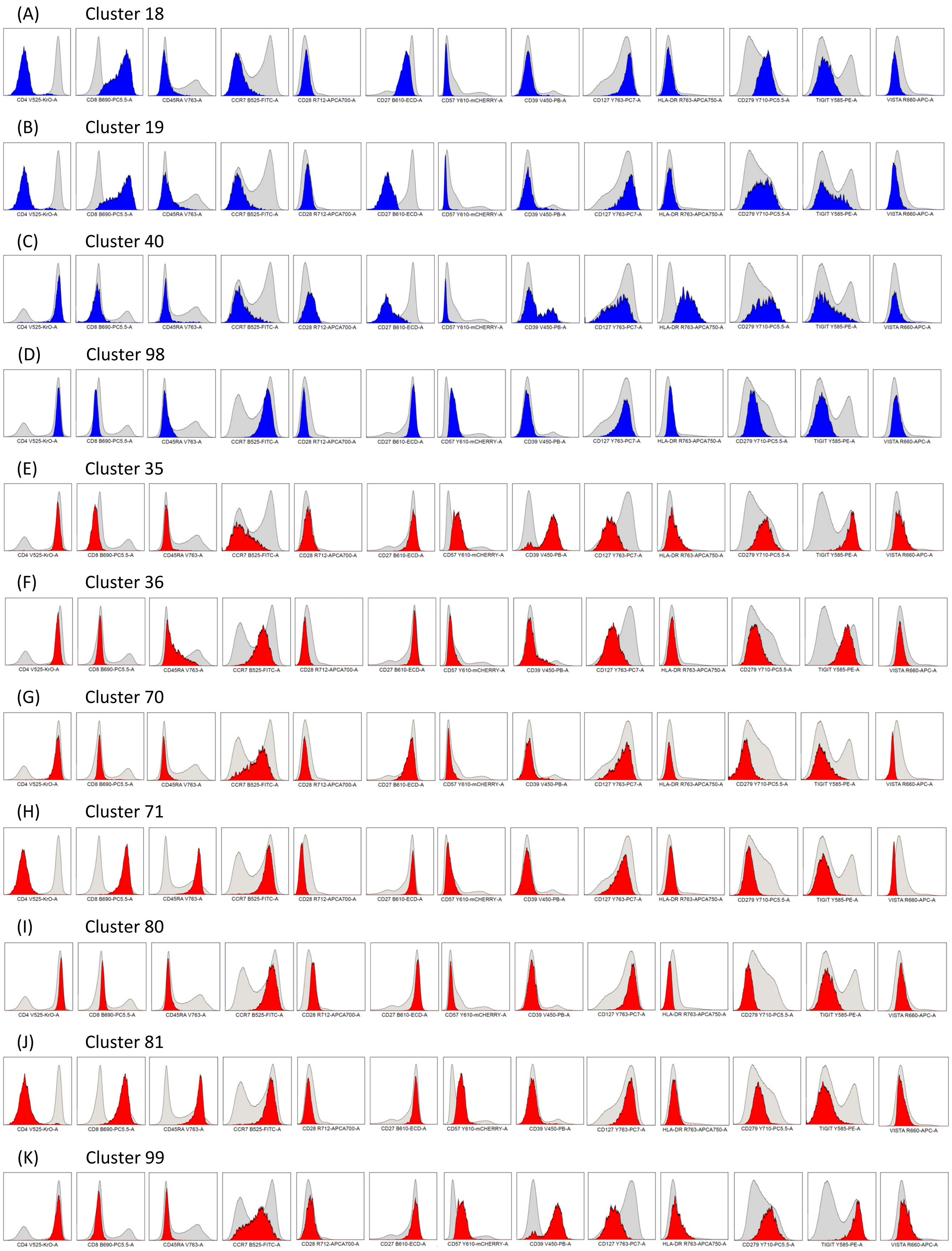

Figure S4 <u>Phenotypic definition of omental fat T cells over- or under-represented in PC patients</u>. Overlay plots comparing manually gated T cells (blue = underrepresented, red = overrepresented) and all other ungated cells (grey). Level of expression for every single marker.
